# Efficacy of copper surfaces in the healthcare environment: a systematic review

**DOI:** 10.1186/2047-2994-4-S1-P45

**Published:** 2015-06-16

**Authors:** S Chyderiotis, C Legeay, D Verjat-Trannoy, F Le Gallou, P Astagneau, D Lepelletier

**Affiliations:** 1 North France healthcare infection control centre, CCLIN Paris Nord, France; 2 Unité de Prévention et de Lutte contre les Infections Nosocomiales, CHU d'Angers, France; 3 North France healthcare infection control centre, CCLIN Paris-Nord, France; 4 Laboratoire de bactériologie-virologie hygiène hospitalière, CHU de Nantes, France; 5 Unité de gestion du risque infectieux, CHU de Nantes, France

## Introduction

To this date, the efficacy of copper in environmental surfaces surrounding patients to decrease bacterial load and ultimately healthcare-associated infections remains controversial.

## Objectives

To determine the potential of copper surfaces to help fight against infection risk in healthcare settings, we conducted a systematic review.

## Methods

A PubMed and Nosobase (French database on infection control) search was performed by two investigators with the following key words: Copper; Surface; Anti-infective; Antimicrobial; Activity. Both in vitro and in vivo studies were included, studies assessing the effects of copper outside of hospital surfaces were excluded (water, air). English, French and Spanish languages were included based on investigators’ skills. Studies encountered on Nosobase and published in non-indexed reviews were excluded.

## Results

A total of 3,289 articles were retrieved with those keywords. Based on titles and after reading abstracts and articles, the investigators selected 34 articles including 24 in vitro studies and 10 in vivo (on-site environmental or clinical) studies.

In vitro studies mostly demonstrated a broad-spectrum activity of copper with a significant decrease of antimicrobial load on copper surfaces compared to control surfaces (mainly stainless steel and PVC). In vivo studies assessed the total flora reduction as the main outcome with an important decrease on most copper surfaces compared to controls.

An important heterogeneity in the design and the results of these studies was observed, making extrapolations to a clinical impact of copper surfaces difficult.

One study in particular assessed the nosocomial infections and/or colonisation with MRSA or VRE as the outcome after introducing 6 copper objects in ICU rooms using a randomized controlled trial. The authors observed a 50% decrease in healthcare-associated infections, although several methodological issues could be addressed regarding the study design.

## Conclusion

Although copper surfaces gained much interest during the past few years, there is a lack of clinical studies to demonstrate a significant effect on patient outcome. Cost-effectiveness studies should also be conducted before concluding on the benefits of copper surfaced equipment for healthcare settings.

## Disclosure of interest

None declared.

